# Significance of Platelet Count in Esophageal Carcinomas

**DOI:** 10.4103/1319-3767.77245

**Published:** 2011

**Authors:** Ali Aminian, Faramarz Karimian, Rasoul Mirsharifi, Abbas Alibakhshi, Habibollah Dashti, Yosra Jahangiri, Saeed Safari, Hamid Ghaderi, Morteza Noaparast, Sharareh M. Hasani, Alireza Mirsharifi

**Affiliations:** Department of General Surgery, Tehran University of Medical Sciences, Tehran, Iran

**Keywords:** Cancer, esophagus, platelet, prognosis, thrombocytosis

## Abstract

**Background/Aim:**

Thrombocytosis is found to be associated with unfavorable prognosis in esophageal carcinoma. Platelets produce thymidine phosphorylase which is a platelet-derived endothelial cell growth factor with angiogenic activity. Increased platelet count may be translated into enhanced tumor growth. We examined the relation between platelet count and several prognostic variables in patients with esophageal cancer.

**Patients and Methods:**

Three hundred and eighty-one cases with esophageal cancer that underwent esophagectomy in a referral cancer institute during a 5-year period were studied retrospectively. The relation between preoperative platelet count and patient age, gender, site of tumor, presence of multiple cancers and clinicopathological characteristics including histological type, tumor size, depth of penetration (T), lymph node involvement (N), distant metastasis (M), degree of differentiation, presence of vascular, lymphatic and perineural invasion was examined.

**Results:**

Squamous cell carcinoma (SCC) constituted 93% and adenocarcinoma 7% of cases. Most of patients were in stage III, followed by stage II. The mean platelet count was 245±76 (× 10^9^ /L). There was no statistically significant correlation between platelet counts with prognostic factors except a weak linear correlation between platelet count and and tumor size (*P*= 0.03, Pearson correlation coefficient: 0.16). Patients with adenocarcinoma had a higher platelet count than those with SCC (*P*= 0.003).

**Conclusion:**

Platelet count does not correlate with prognostic factors in esophageal cancer. However, it is significantly different between SCC and adenocarcinoma of esophagus.

Throughout the world, cancer of esophagus is the sixth most common cause of cancer-related mortality.[1,2] Esophageal cancer is endemic in some regions of Iran.[3–5]

Many factors in patients with malignancy influence prognosis. High platelet count and thrombocytosis is seen in some patients with cancer.[6–8] Platelet count is inversely related to prognosis in various cancers; high count is associated with poor prognosis.[9,10] Whether thrombocytosis precedes malignant changes or follows it is not clear. Significance of platelet count in carcinoma of esophagus has been studied previously. High platelet counts were found to be associated with tumor progression and poor survival in esophageal cancer.[11] Platelets are the source of thymidine phosphorylase (TP). TP is known to be a platelet-derived endothelial cell growth factor which has potent angiogenic activity.[12] This substance is expressed at higher levels in a wide variety of solid tumors compared to normal tissues.[13] Increased TP expression and activity was found to be associated with poor prognosis in various solid tumor tissues.[14] Several different angiogenic factors produced by tumor cells and host cells may regulate angiogenesis during different steps of esophageal carcinogenesis. Findings suggest that angiogenic trigger is an early event in the development of invasive carcinoma.[15] High levels of TP are associated with increased micro-vascular density that facilitates tumor progression. All these factors, cumulatively, lead to poor prognosis in esophageal squamous cell carcinoma (SCC).[16,17] In this study, we investigated the relation between platelet count and prognostic factors in patients with esophageal cancer.

## PATIENTS AND METHODS

After institutional board review approval, all cases with esophageal cancer that underwent esophagectomy in a referral cancer institute in Tehran, Iran during a 5-year period (January 2002-December 2006) were studied retrospectively. Tumors located in the cervical esophagus and subcardia as well as administration of neoadjuvant therapy was considered as exclusion criteria of this study. Patients with lower esophageal tumor underwent transhiatal resection (Orringer technique) and those with mid- and upper-third esophageal tumor had transthoracic esophagectomy (Ivor-Lewis or Mckeown procedures).

Data were collected retrospectively from the patients’ charts. The following information was recorded: preoperative platelet count (× 10^9^ /L), patient age, gender, site of tumor, presence of multiple cancers and clinicopathological characteristics including histological type, tumor size, depth of penetration (T according to TNM classification), lymph node involvement (N according to TNM classification), distant metastasis (M according to TNM classification), degree of differentiation, presence of vascular, lymphatic and perineural invasion. Since there were a limited number of cases in each T_1_ and T_2_ subgroups, the cases were classified as less penetrating tumors (T_1_ and T_2_) and more penetrating tumors (T_3_ and T_4_).

Platelet counts were presented as the mean ± standard deviation (×10^9^ /L). The relation between platelet count and clinicopathological characteristics was assessed with Chi-square, student’s t and Pearson correlation tests. In all cases the probability of type 1 error <0.05 was taken as the criterion of significance. Data handling and analysis were performed with SPSS software for Windows, version 11 (SPSS Inc., Chicago, IL).

## RESULTS

Three hundred and eighty-one cases with esophageal cancer were included. The mean age was 62.8±11.7 years. SCC constituted 93% and adenocarcinoma 7% of cases. Most of patients were in stage III, followed by stage II. Most tumors were located in the lower part of esophagus (60%). The details of clinicopathological findings were summarized in Tables 1 and 2.

**Table 1:** Platelet count and clinicopathological variables in esophageal carcinoma

Variable	N (%)	Platelet count (×10^9^/L)	*P* value
Gender			0.100
Male	201 (53)	239±69	
Female	177 (47)	252±84	
Type			0.003
Squamous cell carcinoma	331 (93)	241±73	
Adenocarcinoma	24 (7)	290±111	
Location			0.501
Upper thoracic	6 (2)	263±99	
Middle thoracic	127 (38)	238±71	
Lower thoracic	201 (60)	247±80	
Degree of differentiation			0.803
Well	101 (31)	248±85	
Moderate	164 (50)	246±76	
Poor	63 (19)	240±71	
Lymphatic invasion			0.756
Present	120 (43)	244±80	
Absent	157 (57)	244±73	
Vascular invasion			0.116
Present	134 (46)	252±85	
Absent	157 (54)	237±71	
Perineural invasion			0.173
Present	67 (25)	232±62	
Absent	203 (75)	245±80	
Multiple cancers			0.205
Present	10 (3)	274±126	
Absent	324 (97)	243±75	

**Table 2:** Platelet count and TNM classification in esophageal carcinoma

Variable	N (%)	Platelet count (×10^9^/L)	*P* value
Depth of penetration			0.474
T1 and T2	70 (22)	239±63	
T3 and T4	246 (78)	246±77	
Lymph nodes metastasis			0.356
Positive	152 (45)	251±83	
Negative	187 (55)	243±72	
Distant metastasis			0.756
Present	16 (5)	253±106	
Absent	306 (95)	245±75	

The mean platelet count was 245±76 (×10^9^ /L). There was no statistically significant correlation between platelet counts with gender, site of tumor, degree of penetration, lymph node involvement, distant metastasis, degree of differentiation, presence of vascular, lymphatic and perineural invasion and presence of simultaneous multiple cancers [Tables1 and 2].

The platelet count in patients with adenocarcinoma was significantly higher than patients with SCC, with a mean difference of 48±16 (×10^9^ /L) (*P*= 0.003) [Table 1]. The grade and stage of tumors were not statistically different between SCC and adenocarcinoma histology [Table 3].

**Table 3:** Comparison of clinicopathological variables between squamous cell carcinoma and adenocarcinoma of esophagus

Variable	Squamous cell carcinoma	Adenocarcinoma	*P* value
Depth of penetration			0.754
T_1_ and T_2_	62 (22)	4 (25)	
T_3_ and T_4_	224 (78)	12 (75)	
Lymph nodes metastasis			0.146
Positive	130 (43)	12 (60)	
Negative	170 (57)	8 (40)	
Distant metastasis			0.205
Present	12 (4)	2 (11)	
Absent	272 (96)	17 (89)	
Degree of differentiation			0.061
Well	90 (31)	8 (40)	
Moderate	149 (51)	5 (25)	
Poor	51 (18)	7 (35)	
Lymphatic invasion			0.288
Present	105 (43)	8 (57)	
Absent	141 (57)	6(43)	
Vascular invasion			0.389
Present	115 (45)	10 (56)	
Absent	140 (55)	8 (44)	
Perineural invasion			0.510
Present	57 (24)	5 (31)	
Absent	181 (76)	11 (69)	

Figures in parentheses are in percentage

**Figure 1:**
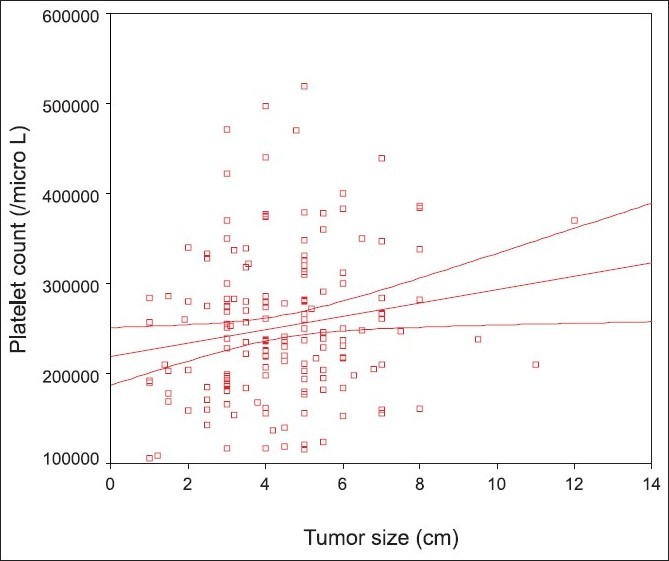
Correlation between platelet count and tumor size

There was a weak linear correlation between platelet count and tumor size (*P* = 0.03, Pearson correlation coefficient: 0.16) [Figure 1].

## DISCUSSION

We defined thrombocytosis as a platelet count greater than 400 (×10^9^ /L).[8] In this regard, only 3.4% of our patients had thrombocytosis. In fact the mean platelet count of patients in this study was near to lower limit of normal range. Previous studies have shown that both thrombocytosis and thrombocytopenia are associated with unfavorable prognosis.[6–10] According to one study, patients with TP-positive esophageal tumors have a poorer prognosis than those with TP-negative tumors. Additionally, TP inhibition suppresses tumor growth by increasing the proportion of apoptotic cells and probably inhibiting angiogenesis. Absence of thrombocytosis may be interpreted as TP-negative tumor (inhibition of TP expression) leading to a better prognosis.[18] Absence of thrombocytopenia is also shown to be associated with better prognosis in patients with esophageal SCC without tumor obstruction.[19] However, our patients were not thrombocytopenic either.

Platelet count in our series showed no significant association with major prognostic variables for esophageal cancer including gender, site of tumor, degree of penetration, lymph node involvement, distant metastasis, degree of differentiation, presence of vascular, lymphatic and perineural invasion and presence of simultaneous multiple cancers; except for a weak linear correlation with tumor size. This finding is likely in favor of TP-negative tumors (absence of TP expression) being the dominant pattern in our patients. Studies indicate that platelet counts are significantly increased in patients with large tumors, deep tumors, those with nodal involvement and patients with distant metastasis.[11] However, there are also reports indicating that higher TP levels expressed by esophageal tumors do not correlate with the histopathological grading of tumor, depth of tumor invasion and lymph node metastasis.[20] We did not measure TP levels in our patients so we cannot determine whether they were TP-negative or TP-positive but with no correlation.

We found that patients with adenocarcinoma of esophagus had a significantly higher platelet count compared to those with SCC (*P* = 0.003), in the presence of similar stage and grade of tumors [Table 3]. We could not find any similar report. Adenocarcinoma developing at other sites including kidney, colon and female reproductive organs express thrombocytosis.[7,8,10] A high preoperative platelet count was an independent prognostic variable for mortality in patients with adenocarcinoma of colon and rectum.[8] The correlation of higher platelet counts, in adenocarcinoma of esophagus, with prognosis must be defined in further studies. The clinical significance of this finding with regard to therapeutic decisions may then be decided.

The main limitation of this study was gathering of data retrospectively. Another important issue was that we did not measure TP level. The high incidence of SCC (93%) in comparison to adenocarcinoma (7%) may have had an adverse effect on statistical analysis.

It may be concluded that platelet count may not always correlate with prognostic variables in SCC of esophagus. However, platelet count may be significantly higher in adenocarcinoma of esophagus, as found in this study.
